# Imatinib (STI571) Inhibits the Expression of Angiotensin-Converting Enzyme 2 and Cell Entry of the SARS-CoV-2-Derived Pseudotyped Viral Particles

**DOI:** 10.3390/ijms22136938

**Published:** 2021-06-28

**Authors:** You-Zhe Lin, Yi-Chun Shen, Wan-Rong Wu, Wei-Jan Wang, Yuan-Liang Wang, Chen-Yuan Lin, Mien-Chie Hung, Shao-Chun Wang

**Affiliations:** 1 Graduate Institute of Biomedical Sciences, College of Medicine, China Medical University, Taichung 40402, Taiwan; yaweyoung@gmail.com (Y.-Z.L.); u104076012@cmu.edu.tw (Y.-C.S.); happy9827000@gmail.com (W.-R.W.); yuanliang0402@gmail.com (Y.-L.W.); 2 Research Center for Cancer Biology, China Medical University, Taichung 40402, Taiwan; cvcsky@mail.cmu.edu.tw; 3 Department of Biological Science and Technology, China Medical University, Taichung 40402, Taiwan; 4 Center for Molecular Medicine, China Medical University Hospital, Taichung 404332, Taiwan; 5 Department of Hematology and Oncology, China Medical University Hospital, Taichung 404332, Taiwan; jnynlin@mail.cmu.edu.tw; 6 Department of Biotechnology, Asia University, Taichung 41354, Taiwan; 7 Department of Cancer Biology, University of Cincinnati, Cincinnati, OH 45267, USA

**Keywords:** COVID-19, ACE2, imatinib, tyrosine kinase

## Abstract

A group of clinically approved cancer therapeutic tyrosine kinase inhibitors was screened to test their effects on the expression of angiotensin-converting enzyme 2 (ACE2), the cell surface receptor for SARS-CoV-2. Here, we show that the receptor tyrosine kinase inhibitor imatinib (also known as STI571, Gleevec) can inhibit the expression of the endogenous ACE2 gene at both the transcript and protein levels. Treatment with imatinib resulted in inhibition of cell entry of the viral pseudoparticles (Vpps) in cell culture. In FVB mice orally fed imatinib, tissue expression of ACE2 was reduced, specifically in the lungs and renal tubules, but not in the parenchyma of other organs such as the heart and intestine. Our finding suggests that receptor tyrosine kinases play a role in COVID-19 infection and can be therapeutic targets with combined treatments of the best conventional care of COVID-19.

## 1. Introduction

The COVID-19 pandemic, which is caused by the novel coronavirus SARS-CoV-2, has impacted not only healthy individuals, but more importantly, people with comorbidity medical conditions such as diabetes, lung diseases, and cancer [1]. SARS-CoV-2 infection poses a significant threat to cancer patients, and the associated morality seems to be determined by age, gender, and comorbidities, but the potential relationships of patients’ anti-cancer treatments with the susceptibility of virus infection remain elusive [2]. The SARS-CoV-2 virus enters the cells through the action of angiotensin-converting enzyme 2 (ACE2), the major receptor on the cell surface for the protease-processed spike protein of the virus during cell entry [3,4]. Tyrosine kinase inhibitors (TKIs) are the major medicines of targeted therapy for cancer treatment; however, their impacts on COVID-19 infection are not well understood. As an initial effort to understand the potential influence of cancer treatments on SARS-CoV-2 infection, the current study tested a panel of FDA-approved TKIs for their effects in viral infection. We chose to begin by assessing the regulation of ACE2 expression in vitro and in vivo and examining the cell entry activity of the SARS-CoV-2 pseudoviral particles.

Imatinib (STI571, Gleevec) is a TKI with multiple targets, including c-ABL, PDGFR, c-KIT, and CSF1R [5]. It has been reported that, with in vitro infection assays, imatinib inhibits the infection of other coronaviruses, including SARS-CoV and MERS-CoV, the causes of the 2003 severe acute respiratory syndrome (SARS) pandemic and the 2012 Middle East respiratory syndrome (MERS), respectively [6]. It has therefore been speculated that imatinib may also be effective for COVID-19 treatment. Despite the enthusiasm surrounding imatinib’s possible application for treating COVID-19 infection, the potential action mechanisms of the drug in inhibiting viral activities remain to be determined. In this report, we show that imatinib treatment inhibits ACE2 expression in multiple cell lines and inhibits the entry of SARS-CoV-2-based pseudoviral particles. Mice fed with imatinib exhibit downregulation of ACE2 in a tissue-specific manner.

## 2. Results

### 2.1. Imatinib Repressed the Reporter Activity Driven by the ACE2 Promoter

**Figure 1:**
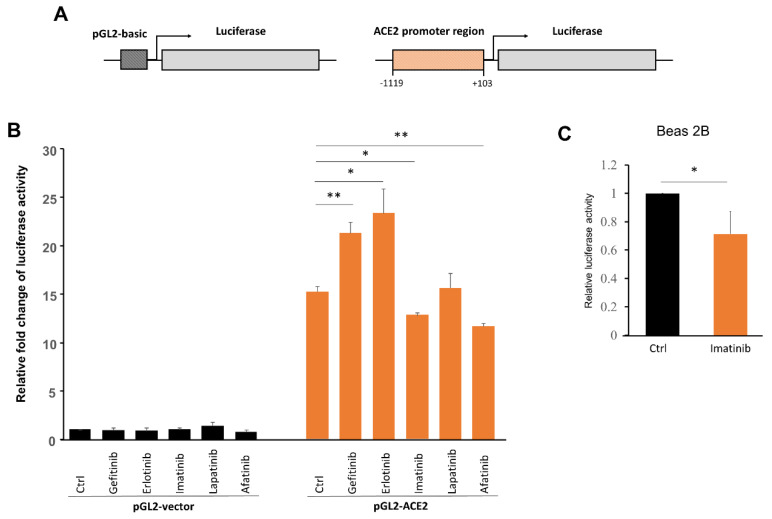
Imatinib inhibited the reporter activity driven by the ACE2 promoter. (**A**) Schematic illustration of the ACE2-luc reporter construct (ACE2(−1119)-luc). (**B**) The reporter plasmid ACE2(−1119)-luc or the backbone control vector (pGL2) was transfected into 293T cells, followed by treatments with the indicated drugs for 24 h with doses of gefitinib (2.5 μM), erlotinib (2.5 μM), lapatinib (2.5 μM), or imatinib (2.5 μM). The luciferase activity was then measured and plotted. Bars, standard deviation; * *p* < 0.05; ** *p* < 0.01; the p-values were determined by Student’s *t*-test. (**C**) Beas 2B cells were transfected with ACE2(−1119)-luc, treated with and without imatinib (at 10 μM for 24 h), followed by the analysis of luciferase activities as described in (**B**). Bars, standard deviation; * *p* < 0.05 determined by Student’s *t*-test.

A reporter system of the ACE2 promoter was used to assess the effects of a panel of TKIs on the promoter activity in which the luciferase reporter gene was driven by the proximal ACE2 promoter (−1119 to +103 nt [7]) (Figure 1A). The reporter plasmid was transiently transfected into the human embryonic kidney cells HEK293, which were then treated with different TKIs, including the epithelial growth factor receptor (EGFR) inhibitors gefitinib, erlotinib, lapatinib, and afatinib, as well as imatinib. The results showed that only imatinib and afatinib suppressed the reporter activity by 16% and 23%, respectively (Figure 1B). It should be noted that although gefitinib, erlotinib, lapatinib, and afatinib can all inhibit the EGFR, only afatinib treatment resulted in inhibition of the reporter activity. This suggests a non-specific, EGFR-independent mechanism of afatinib. Imatinib also inhibited the ACE2 reporter activity in HEK293 cells, and the inhibitory activity was further validated by conducting the reporter assay in the human non-tumorigenic lung epithelial Beas 2B cells (Figure 1C). Based on the ACE2 reporter results, we decided to focus on imatinib for further characterization.

### 2.2. Imatinib Treatment Suppressed Endogenous ACE2 Expression in Multiple Cell Lines

**Figure 2:**
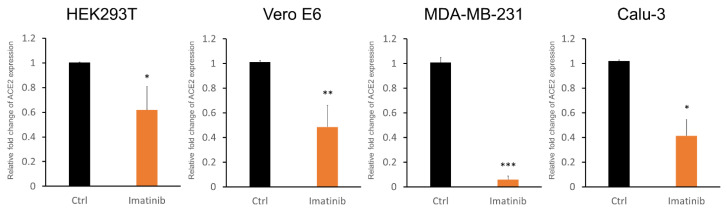
Downregulation of the endogenous ACE2 gene by imatinib treatment. The indicated cell lines were mock-treated or treated with imatinib (at 5 μM for 24 h). The mRNA expression of the endogenous ACE2 gene was measured by qRT-PCR. For each cell line, the data obtained from three independent experiments were plotted. Bars, standard deviation. The *p*-values determined by Student’s *t*-tests are indicated by asterisks. * *p* < 0.05; ** *p* < 0.01; *** *p* < 0.001.

**Figure 3:**
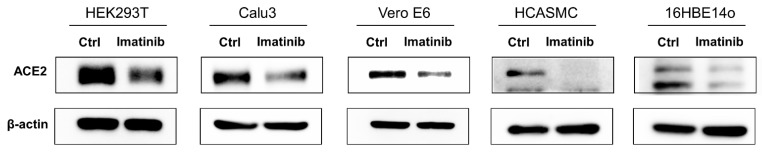
Treatment with imatinib suppressed ACE2 protein expression in multiple cell lines. Cell lysates of the indicated cell lines treated with and without imatinib (at 5 μM for 24 h) were isolated and analyzed by Western blotting using an anti-ACE2 polyclonal IgG. The expression of β-actin was used as the internal equal loading control.

The effects of imatinib on the expression of the endogenous ACE2 gene in multiple cell lines derived from COVID-19-relevant organs, including HEK293 (human kidney), Vero E6 (African green monkey kidney epithelial), and Calu3 (human lung adenocarcinoma), were assessed. The MDA-MB-231 cells derived from human breast carcinoma were also included because ACE2 functions in this cancer cell line have been reported [8]. Cells were treated with and without imatinib, and the cellular ACE2 mRNA expression was measured by quantitative reverse transcription–polymerase chain reaction (qRT-PCR). The results showed that treatment with imatinib did downregulate RNA expression of the ACE2 gene in these cell lines (Figure 2). The decrease in exogenous promoter reporter activity and the endogenous RNA expression of ACE2 by imatinib suggested that imatinib should also be able to repress the protein expression of ACE2. Indeed, Western blotting analysis showed that treatment with imatinib resulted in downregulation of the ACE2 protein in multiple cell lines, including HEK293, Calu3, and Vero E6, as well as two cell lines derived from the normal endothelium and respiratory epithelium: the HCASMCs (human primary coronary artery smooth muscle cells) and 16HBE14o (human bronchial epithelial) cells, respectively (Figure 3). These results demonstrated the activity of imatinib in inhibiting ACE2 expression in cells of different tissue types and origins.

### 2.3. Imatinib Inhibited Cell Entry of the SARS-CoV-2 Pseudotype Particles

**Figure 4:**
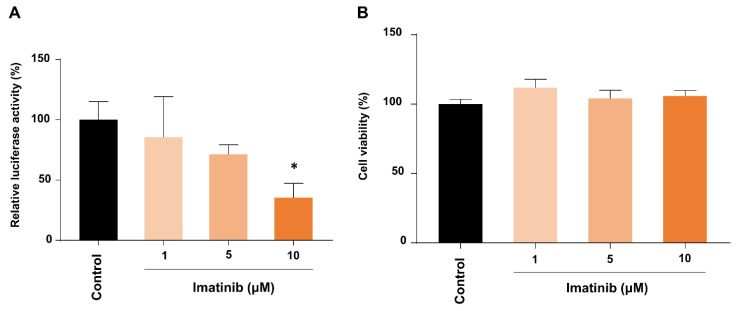
Imatinib inhibited the cell entry of SARS-CoV-2 pseudotype particles. (**A**) Vero E6 cells were pre-incubated with the indicated doses of imatinib for 24 h and then inoculated with the pseudotyped viral particles of SARS-CoV2-S virus; the viral entry was assessed by measuring the virus-encoded luciferase activity in cell lysates. The relative entry efficiency was quantitated by normalization with the efficiency of the untreated cells. Bars, standard deviation; * *p* < 0.05 determined by Student’s *t*-test. For each treatment, three biological repeats with quadruplicate samples were conducted. (**B**) Cell viability was measured using the Cell Titer Glo assay (Promega). Bars, standard deviation.

We then hypothesized that ACE2 suppression by imatinib could be translated to the inhibition of SARS-CoV-2 infection by the drug. To test this possibility, pseudotype viral particles of SARS-CoV-2 were used in a luciferase-based cell entry assay in the simian kidney epithelial Vero E6 cells at a multiplicity of infection (MOI) of about 0.28. Treatment with imatinib inhibited cell entry of the pseudovirus in a dose-dependent manner up to 10 μM (Figure 4A), while there was no effect on cell viability under the same conditions (Figure 4B). Thus, these data suggest that imatinib treatment can effectively prevent SARS-CoV-2 infection with low toxicity to cells.

### 2.4. Imatinib Treatment Reduced the Expression of ACE2 in a Tissue-Specific Manner

**Figure 5:**
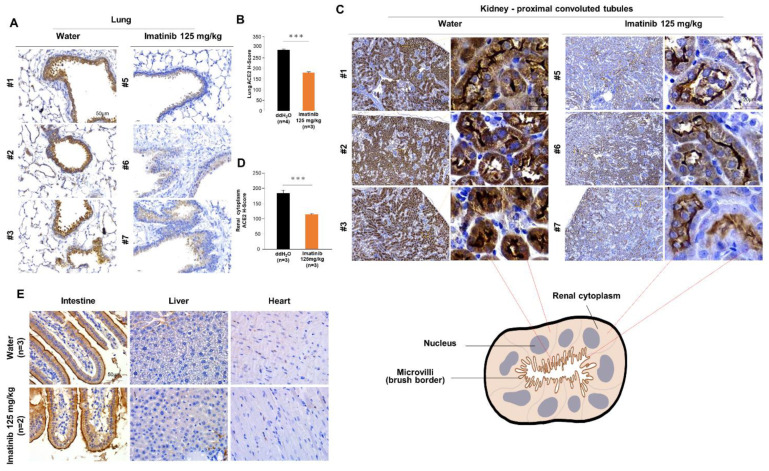
Imatinib treatment reduced the expression of ACE2 in a tissue-specific manner. FVB mice were fed with imatinib or water for one week, and the organs were isolated and processed for analysis by IHC of ACE2. ACE2 staining was assessed by Allred scoring for the lungs (**A**,**B**), the kidneys (**C**,**D**), and other organs, including the intestine, liver, and heart (**E**). In (**C**), the boxed areas are magnified to show the detailed structures of the proximal convoluted tubules. Bars, standard deviation; *** *p* < 0.001 determined by Student’s *t*-test.

To determine whether the downregulation of ACE2 by imatinib could be replicated in vivo, mice were fed daily with imatinib (125 mg/kg) or water (the solvent) as the mock treatment control by oral gavage for one week before being sacrificed (Supplementary Figure S1A). Different organs were dissected, and the expression of ACE2 was assessed by immunohistochemical staining (IHC) using the anti-ACE2 polyclonal IgG. We observed a significant downregulation of ACE2 expression in the lungs (Figure 5A,B) and the cytoplasm of proximal convoluted tubules (PCTs) in the kidneys (Figure 5C,D) of imatinib-treated mice compared to the mock-treated control mice. The PCTs are nephron tissues with functions such as the resorption of electrolytes, water, sugar, and nutrients from the glomerular filtrates. The apical membrane of epithelial cells lining the PCTs, known as the brush border, is composed of striated microvilli, which are responsible for the resorption process and express high levels of membrane ACE2. It should be noted that the downregulation of ACE2 by imatinib in the kidneys was visible mainly in the cytoplasm of the renal cells but not in the apical brush border, probably due to the prominent ACE2 expression in the brush border (Supplementary Figure S1B). It should also be noted that the imatinib-mediated downregulation of ACE2 appeared to be tissue-specific; ACE2 expression was not altered by imatinib treatment in the intestinal epithelium, liver, or heart. Overall, our results corroborate an anti-COVID-19 function of imatinib through the inhibition of ACE2 expression in an organ-specific manner.

## 3. Discussion

The current study suggests that the tyrosine kinase inhibitor imatinib can inhibit cell entry of SARS-CoV-2 through downregulation of the spike protein receptor ACE2. ACE2 is a central member of the renin–angiotensin axis, which converts angiotensin I to angiotensin II [9,10]. Angiotensin II is the key biological peptide of the axis driving vasocontraction, inflammation, fibrosis, apoptosis, and eventually, acute lung injury [10]. ACE2 cleaves the angiotensin II peptide and protects the tissues through vasodilation, proliferation suppression, and anti-fibrosis [11]. It has been reported that ACE2 inhibits angiogenesis and metastasis by decreasing the activity of the tyrosine kinase vascular endothelial growth factor receptor (VEGFR) pathway [8]. Whether and how ACE2 cross-talks with the tyrosine kinase receptors remains largely unexplored.

Prior studies have suggested the potential of imatinib in anti-COVID-19 activity. Imatinib is a multi-target inhibitor of multiple RTKs, including c-ABL, c-KIT, platelet-derived growth factor receptor (PDGFR), and VEGFR [12,13]. Whether imatinib exerts its anti-COVID activity through these molecular targets or by other off-target mechanisms remains to be determined. Regardless of the mechanisms, our results, together with prior studies, suggest that the kinase inhibition activity of imatinib may hinder viral entry or propagation. As well as the suppression of viral infection, it has been proposed that imatinib can also modulate inflammatory responses by suppressing the pro-inflammatory cytokine receptor and indirectly ameliorate the respiratory syndrome, in particular, the pulmonary infiltration [1]. It has also been reported that imatinib treatment suppresses inflammatory responses by attenuating the secretion of cytokines such as IL-6 and IL-8, as well as inhibiting NF-κB and AP1, the key transcription factors involved in immune responses [14]. Thus, these studies suggest that imatinib treatment can counteract the COVID-19 virus through the inhibition of viral infection and amelioration of the associated acute respiratory inflammation syndrome. This expectation is supported by a case report in which a hospitalized COVID-19 patient developed radiological deterioration under standard treatment protocols, although recovered after the addition of imatinib to the treatment regimen [15]. A multi-center survey of more than 7000 chronic myeloid leukemia patients showed that the incidence of COVID-19 infection was extremely low among the patients treated with tyrosine kinase inhibitors, including imatinib [16]. With this perspective, three clinical trials have been initiated to test the safety and efficacy of imatinib for hospitalized COVID-19 patients in different countries, including the Netherlands (EudraCT 2020-001236-10), France (NCT04357613/phase 2), and the United States (NCT04394416/phase 3 [17]). The results of these trials were not available at the time of writing this article. On the other hand, however, one laboratory study by Zhao et al. has reported that imatinib is not effective in the inhibition of viral entry and replication of SARS-CoV-2 in the colon cancer cell line Caco-2 [18]. The reason for the discrepancy is not clear, but the assay methods and experimental conditions may play a role. Zhao et al. used GFP as the reporter, whereas the current study used a reporter system based on luciferase. In addition, the multiplicity of infection (MOI) used in the viral pseudoparticle entry assay was about 0.002 by Zhao et al. and about 0.28 in the current study, which is a considerable difference. More importantly, the current study used the kidney epithelial cell line Vero E6 instead of the Caco-2 colon cancer cell line used in the study by Zhao et al. Thus, it is conceivable that the different outcomes may stem from the different experimental conditions used in these two studies.

In the current report, we have shown that imatinib inhibited the RNA and protein expression of ACE2 in multiple cell lines of different species and organ types, including lung and kidney tissues in vitro and in vivo. This is in correlation with the observation that imatinib inhibited the cell entry of the pseudoviral particles of SARS-CoV-2. The expression of ACE2 in the lungs and kidneys was suppressed by imatinib, suggesting that cancer patients receiving the therapy may also benefit with protection from COVID-19 infection. Conversely, our results showed a slight but statistically significant increase in ACE2 by the EGFR inhibitors gefitinib and erlotinib, possibly indicating that cancer patients treated with these drugs could face a higher risk of being infected by the virus. As the pandemic continues to impact the global society, epidemiologic analyses should explore these questions. Furthermore, the current study has not addressed the emerging threats of SARS-CoV-2 variants. These emerging variants appear to be more dangerous than the original strain of the virus and warrant further study [19].

In the Figure 1C of this study, the Western blotting analysis of ACE2 probed by the anti-ACE2 polyclonal IgG showed two bands in the 16HBE14o cells, instead of only one band as demonstrated in all other cell lines tested. The reason for the double band in the cell line is not clear, but it may be caused by an ACE2-derived polypeptide after protein degradation or post-translational processing or a variant of ACE2 isoform specifically expressed in the cell line. It could also be due to a cross-reaction of the antibody with a protein specifically expressed in the cell line. Regardless of the mechanisms, the result supports that imatinib inhibits ACE2 protein expression in multiple cell lines.

We did not observe a significant decrease in ACE2 in the renal brush border. This could be due to the exceptionally high levels of ACE2 expressed in the brush border; therefore, higher doses and/or longer treatment with imatinib may be required to downregulate ACE2 in the brush border. Our data also show that the expression of ACE2 was most sensitive to imatinib treatment in the lungs and the kidneys. Pulmonary and renal syndromes are the major pathogeneses associated with COVID-19 infection; therefore, our findings suggest that imatinib could be a candidate medicine for combined treatments with the best conventional care of COVID-19. Further investigation is warranted to determine whether other drugs with similar target spectra are also effective in inhibiting SARS-CoV-2 infection.

## 4. Materials and Methods

### 4.1. Cell Lines, Plasmids, Chemicals, and Antibodies

HEK-293T and MDA-MB-231 cell lines were cultured in DMEM/F12 supplemented with 10% FBS and 1% PSA; Calu3 and Vero E6 were cultured in Eagle’s minimum essential medium supplemented with 10% FBS and 1% PSA; HCASMC was cultured in Medium 231 supplemented with SMGS, as per the manufacturer’s instructions (Invitrogen); 16HBE14o was cultured in a minimum essential medium supplemented with 10% FBS and 1% PSA in fibronectin/Collagen/BSA ECM-coated flasks. The ACE2 reporter ACE2(-1119)-luc was a gift from Dr. Gerhart Ryffel and purchased from Addgene (#31110) [7]. The following chemicals and antibodies were purchased: ACE2 (Abcam, ab15348), β-actin (Santa Cruz), Trilogy™ (Cell Marque, 920P-06), Biotin-anti-rabbit (Jackson, 111-065-003), the avidin–biotin complex (ABC) kit (Vector, PK-6100), the DAB Substrate kit (Vector, SK-4100), and hematoxylin solution (Leica, #3801522).

### 4.2. Luciferase Reporter Assay

To measure the reporter activity, cells were plated in 24-well plates at 1 × 10^4^ cells/well one day prior to the co-transfection with 0.5 μg of ACE2(-1119)-luc or the control vector (pGourL2), along with 5 ng of renilla using Lipofectamine 2000, following the manufacturer’s instructions (Invitrogen). The next day, cells were treated with 2.5 μM gefitinib, 2.5 μM erlotinib, 2.5 μM imatinib, 2.5 μM lapatinib, 2.5 μM afatinib, or the control for 24 h. The luciferase reporter activity was assessed following the manufacturer’s instructions with a dual-luciferase reporter assay system (Promega), and measured by a luminometer. The raw data are shown in Supplementary Figure S3A,B.

### 4.3. qRT-PCR

Total RNA was extracted by the Direct-zol RNA kit (Zymoresearch) following the manufacturer’s protocol. Reverse transcription was performed in M-MLV reverse transcriptase (Invitrogen). For qRT-PCR, the target cDNA was amplified with specific primers by using SYBR Green master mix (Bio-Rad) and a QuanStudio 5 real-time PCR system. Relative levels were calculated using the comparative C_T_ method. The raw data are shown in Supplementary Figure S3C.

### 4.4. Western Blot Analysis

Cell lysates were prepared in the RIPA buffer (150 mM NaCl, 1% NP40, 0.5% DOC, 0.1% SDS, 50 mM Tris-HCl, pH7.5, 10 μg/mL Aprotinin, 5 mM PMSF, 25 mM NaF and 2 mM Na3VO4). Proteins were separated on acrylamide gels, transferred to a PVDF membrane, and probed with the indicated antibodies. Signals were detected by a chemiluminescence-based detection method by a horseradish peroxidase-conjugated secondary antibody.

### 4.5. Pseudotyped Viral Particle (Vpp) Assay for SARS-CoV-2

The SARS-CoV2-S pseudoviral particles were purchased from the RNAi core of the Academia Sinica, Taiwan. Vero E6 cells were cultured in DMEM containing 10% FBS, 1% PSA and 1X GlutaMAX. Cells were seeded into 96-well plates and pre-treated with different doses of imatinib for 24 h, then inoculated with 100 μL of normal media containing the Vpp (MOI = 0.28). After overnight incubation, cells were fed with fresh media. At about 40 h post-inoculation, cells were lysed with 100 μL of a medium containing 50% Steady-Glo (Promega) at room temperature for 5 min. The transduction efficiency was measured by the quantification of the luciferase activity using an ELISA reader. The cytotoxicity of imatinib to Vero E6 cells was assessed by using the Cell Counting Kit-8 (ab228554, Abcam) following the manufacturer’s protocol. Briefly, 5 × 10^4^ cells per well in 96-well plates were seeded in 100 μL of media and cultured for about 24 h, followed by treatment with different doses of imatinib in 100 μL DMEM/high glucose medium containing 5% FBS for 24 h. CCK-8 solution (10 μL) was added, and the cells were incubated for 3 h. The OD values (460 nm) were measured to represent the cell proliferation ability. All experiments were performed in triplicates, repeated three times. The raw data are shown in Supplementary Figure S3D.

### 4.6. Animal Experiment

Animals were raised under ambient temperatures (20–24 °C) and air humidity (50–70%). Wild-type female mice aged between seven and ten weeks were used. Mice were orally treated with control H_2_O (*n* = 4) or imatinib (125 mg/kg) (*n* = 3) for seven continuous days. The mice were sacrificed on the eighth day, and their organs, including the lungs, kidneys, intestine, liver, and heart, were excised and processed for immunohistochemistry staining.

### 4.7. IHC

Mouse tissues were fixed with 10% formalin. The formalin-fixed paraffin-embedded (FFPE) tissues were sectioned at a thickness of 3 μm. Tissue sections were deparaffinized at 65 °C for 1 h followed by incubation in xylene for 10 min; then, they were washed in a concentration gradient of alcohol and rehydrated in distilled water. Antigen retrieval was performed in 1x Trilogy™ at 110–120 °C in a steamer for 15 min and was allowed to cool down for 20 min. The endogenous peroxidase activity was blocked with 3% H_2_O_2_ in distilled water for 15 min. The slides were then washed with PBS buffer. Tissues were blocked with 5% normal goat serum in PBS for 1 h at room temperature. ACE2 primary antibody (1:500), diluted by 5% normal goat serum in PBS solution, were incubated at 4 °C overnight. The primary antibodies were then removed by washing with PBS buffer. The biotinylated anti-rabbit antibodies, diluted in 5% normal goat serum (1:1000) in PBS, were incubated for 1 h at room temperature. After washing with PBS, the avidin–biotin complex (ABC) was applied to the slides, and they were incubated for 1 h followed by washing with PBS. The complex was then visualized by the DAB reaction and counter-stained with hematoxylin.

### 4.8. IHC Quantification and Statistical Analysis

Histoscores (H-scores) were determined according to different signal intensity levels and the percentage of visible cells. For the H-score assessment, the signal intensities were divided into levels of 0, 1+, 2+, and 3+ under 400× magnification. The total number of cells in each field and the number of cells stained at each intensity were counted. H-score = 1 × (percentage of cells scored as 1+) + 2 × (percentage of cells scored as 2+) + 3 × (percentage of cells scored as 3+). Examples of the staining locations and intensities are shown in Supplementary Figure S2.

## Data Availability

The data that support the findings of this study are available from the corresponding author upon reasonable request.

## Supplementary Materials

The following are available online at https://www.mdpi.com/article/10.3390/ijms22136938/s1.
