# Supplementary material for: Imatinib (STI571) Inhibits the Expression of Angiotensin-Converting Enzyme 2 and Cell Entry of the SARS-CoV-2-Derived Pseudotyped Viral Particles
Source: Int J Mol Sci. 2021 Jun 28;22(13):6938. doi: 10.3390/ijms22136938 (PMC8268654; doi:10.3390/ijms22136938)
Supplement: Supplementary file 1 [file ijms-22-06938-s001.zip › ijms-1235100-supplementary.pdf]

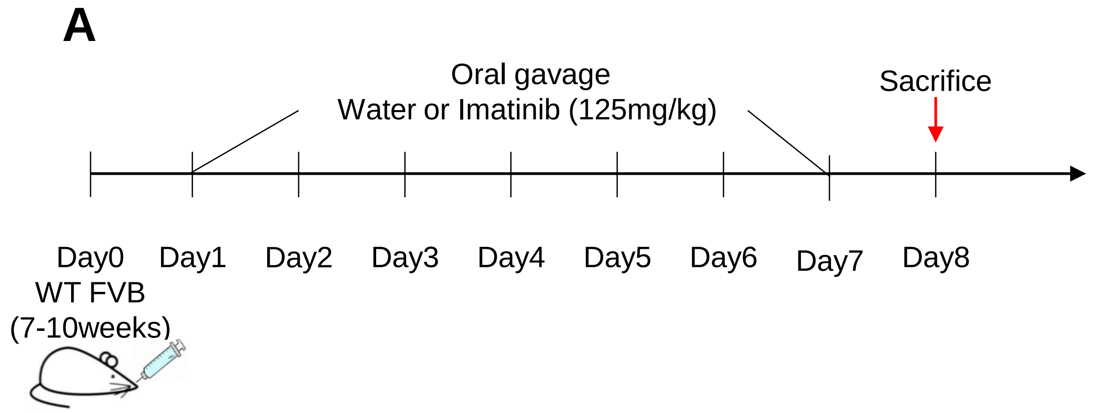

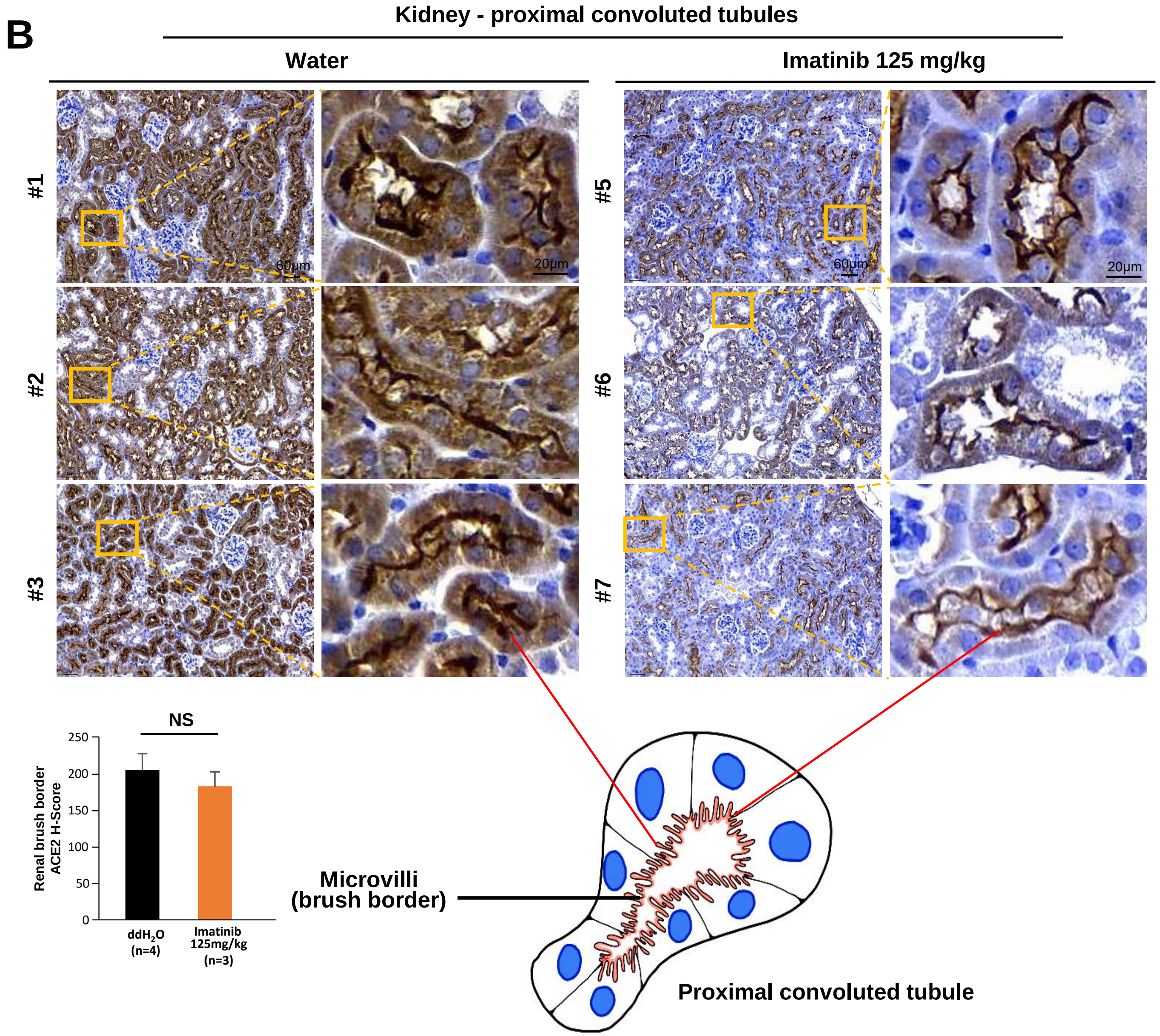

Supplementary Figure S1. Imatinib treatment did not alter the expression of ACE2 in the brush border of the kidneys. A, Schematic diagram of the treatment course. **B**, Kidney proximal convoluted tubules were stained for ACE2 as described in **Figure 5C**. The boxed areas are magnified to show the detail structures of the proximal convoluted tubules. ACE2 staining was assessed by Allred scoring and the data were plotted. Bars, standard deviation; Statistical significance was determined by Student's *t* test.

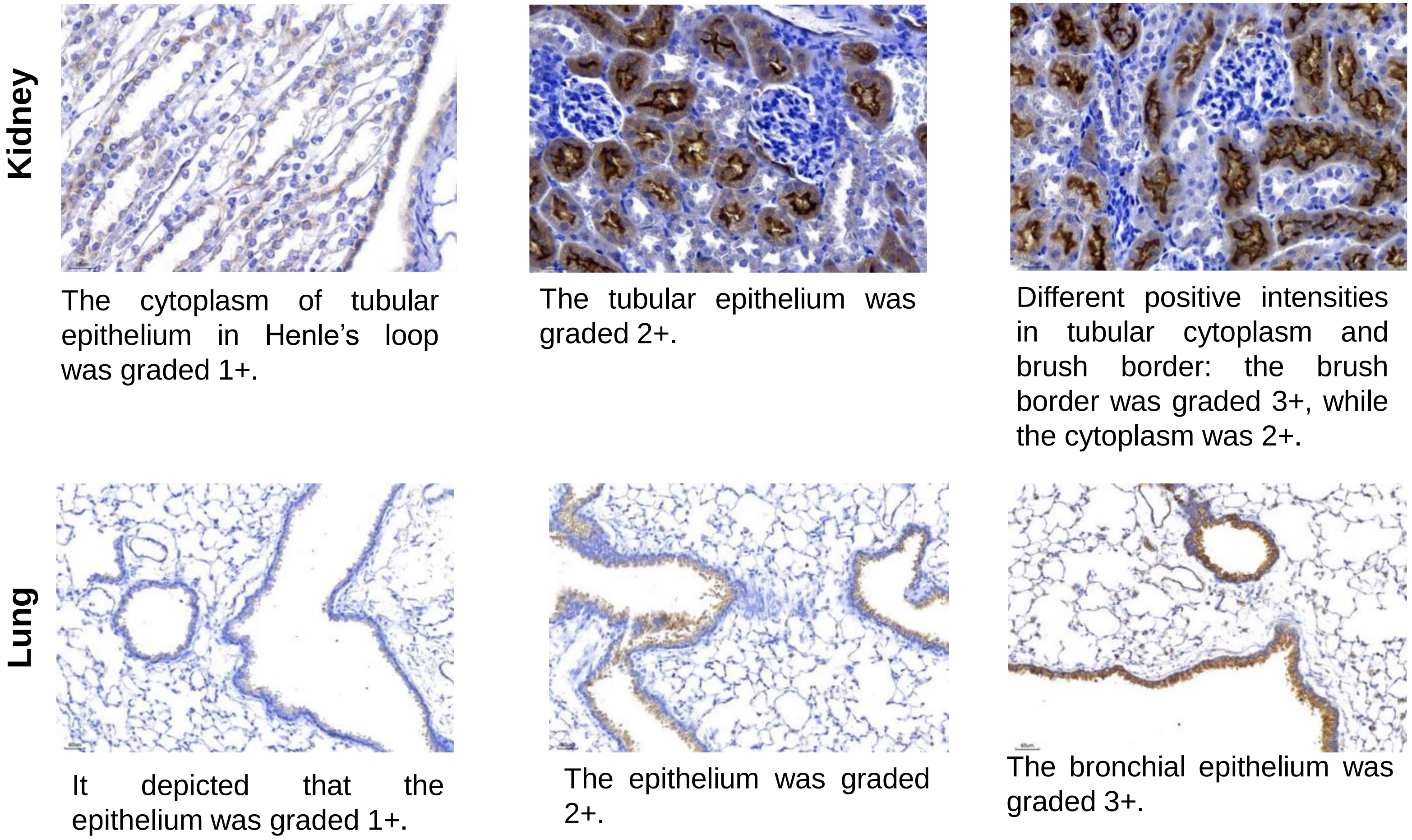

Supplementary Figure S2. Scoring criteria of IHC staining of ACE2 in the kidney and lung tissues. Examples of locations and intensities score are depicted. Only the bronchial epithelium was evaluated. For the detailed procedure and scoring method, refer to the Materials and methods section.

A

		Exp1		Exp2		Exp3	
		**luc**	**renilla**	**luc**	**renilla**	**luc**	**renilla**
**ACE2 -1119**	**DMSO**	48797	6248	38781	5123	63141	7873
	**Gefitinib**	67947	5873	69244	6342	90121	8653
	**Erlotinib**	47185	4326	83017	6782	130928	10214
	**Imatinib**	42582	6532	37696	6112	62298	9333
	**Lapatinib**	51114	5983	60153	8214	103229	12543
	**Afatinib**	23644	4287	45723	7512	70686	11098
**pGL2**	**DMSO**	1767	3212	3236	6471	4239	8653
	**Gefitinib**	2044	3672	2540	5871	4920	9712
	**Erlotinib**	1667	4902	2950	5325	3682	8321
	**Imatinib**	1994	3842	3207	6327	4737	9053
	**Lapatinib**	4301	5021	4088	6421	6653	9832
	**Afatinib**	1188	4523	2161	4852	3454	7543

B

**Beas 2B**	**Exp1**		**Exp2**		**Exp3**	
**ACE2 -1119**	**luc**	**renilla**	**luc**	**Renilla**	**luc**	**renilla**
**DMSO**	1502	2032	1245	2022	915	2025
**Imatinib**	1195	2378	1112	3150	1134	2827

C

**HEK293T**	**Exp1**		**Exp2**		**Exp3**	
	**Ctrl (DMSO)**	**Imatinib**	**Ctrl (DMSO)**	**Imatinib**	**Ctrl (DMSO)**	**Imatinib**
**ACE2 (Ct value)**	28.213	28.787	27.809	28.881	27.350	28.638
**18s (Ct value)**	10.476	10.607	10.460	10.332	10.564	11.791
**Vero E6**	**Exp1**		**Exp2**		**Exp3**	
	**Ctrl (DMSO)**	**Imatinib**	**Ctrl (DMSO)**	**Imatinib**	**Ctrl (DMSO)**	**Imatinib**
**ACE2 (Ct value)**	23.602	24.309	24.163	24.061	25.047	27.159
**18s (Ct value)**	12.905	12.631	13.498	11.887	14.020	15.654
**MDA-MB-231**	**Exp1**		**Exp2**		**Exp3**	
	**Ctrl (DMSO)**	**Imatinib**	**Ctrl (DMSO)**	**Imatinib**	**Ctrl (DMSO)**	**Imatinib**
**ACE2 (Ct value)**	33.379	34.790	32.790	33.653	33.087	34.179
**18s (Ct value)**	13.387	11.718	12.913	8.944	12.684	9.583
**Calu-3**	**Exp1**		**Exp2**		**Exp3**	
	**Ctrl (DMSO)**	**Imatinib**	**Ctrl (DMSO)**	**Imatinib**	**Ctrl (DMSO)**	**Imatinib**
**ACE2 (Ct value)**	30.847	28.791	28.172	30.385	28.971	25.483
**18s (Ct value)**	19.122	15.716	16.348	18.027	17.538	11.730

D

**Vpp assay**	**Ctrl (DMSO)**	**Imatinib 1 uM**	**Imatinib 5 uM**	**Imatinib 10 uM**
**Exp1**	2220	2264	1725	994
**Exp2**	2458	1008	1379	817
**Exp3**	1813	2280	1523	479
**Viability assay**	**Ctrl (DMSO)**	**Imatinib 1 uM**	**Imatinib 5 uM**	**Imatinib 10 uM**
**Exp1**	0.872	0.946	0.926	0.869
**Exp2**	0.875	1.014	0.915	0.916
**Exp3**	0.823	0.913	0.835	0.935

Supplementary Figure S3. Original data of luciferase, qRT-PCR, and Vpp assays. The data derived from Figure 1A (**A**), Figure 1C (**B**), Figure 2 (**C**), and Figure 4 (**D**) are shown. The data of three independent repeats (Exp 1, Exp 2, Exp 3) are shown.
